# Diversity, Virulence, and Clinical Significance of Extended-Spectrum β-Lactamase- and pAmpC-Producing *Escherichia coli* From Companion Animals

**DOI:** 10.3389/fmicb.2019.01260

**Published:** 2019-06-05

**Authors:** Alessio Bortolami, Flavia Zendri, Elena Iuliana Maciuca, Andy Wattret, Christine Ellis, Vanessa Schmidt, Gina Pinchbeck, Dorina Timofte

**Affiliations:** ^1^Department of Veterinary Pathology and Public Health, Institute of Veterinary Science, University of Liverpool, Neston, United Kingdom; ^2^Department of Epidemiology and Population Health, Institute of Infection and Global Health, University of Liverpool, Neston, United Kingdom; ^3^Department of Small Animal Clinical Science, Institute of Veterinary Science, University of Liverpool, Neston, United Kingdom; ^4^Institute of Infection and Global Health, University of Liverpool, Liverpool, United Kingdom

**Keywords:** ESBL, *Escherichia coli*, virulence, antimicrobial resistance, companion animals, United Kingdom

## Abstract

*Escherichia coli* are opportunistic pathogens with the potential to cause a variety of infections in both humans and animals and in many cases have developed antimicrobial resistance. In this study, we characterized extended-spectrum cephalosporin resistant (ESCR) *E. coli* isolates from diseased companion animals (dogs, cats, and horses) and related the results to clinical findings. ESCR *E. coli* clinical isolates obtained over a 6-year period were screened for extended-spectrum β-lactamase (ESBL) and/or plasmid mediated AmpC (pAmpC) and virulence markers likely to be associated with extraintestinal pathogenic *E. coli* (ExPEC). ESBL and/or pAmpC genetic determinants were identified in 79.9% of the ESCR *E. coli* isolates with *bla*_CTX-M_ genes being the most common ESBL genotype of which *bla*_CTX-M-15_, *bla*_CTX-M-14_, and *bla*_CTX-M-55_ were the most prevalent. In addition, *bla*_CMY -2_ was the most common genotype identified amongst pAmpC producing isolates. Phylogenetic group typing showed that B2 was the most prevalent phylogroup among the ESCR *E. coli* isolates, followed by the closely related phylogroups D and F which are also associated with extra-intestinal infections. ESCR was also identified in phylogroups commonly regarded as commensals (B1, A, and C). Virulence factor (VF) scores >2 were mostly present amongst isolates in phylogroup B2. Higher virulence scores were found in isolates lacking ESBL/pAmpC resistance genes compared with those carrying both genes (*p* < 0.05). Five of phylogroup B2 isolates, were typed to the pandemic virulent O25b-ST131 clone and three ST131 isolates carrying *bla*_CTX-M-15_ belonged to the subclade C2/H30Rx whilst one isolate carrying *bla*_CTX-M-27_ typed to the recently described sub-clade C1-M27. MLST typing also identified other sequence types commonly associated with infections in humans (ST410, ST10, and ST648). Most ESCR *E. coli* isolates obtained in pure growth were cultured from normally sterile body sites (mostly from urinary tract infections, UTIs) whilst only a small proportion were obtained from body sites populated with commensal flora (*p* < 0.0001). Our study has shown that ExPEC ESBL/pAmpC producing *E. coli* isolates are common amongst companion animal isolates and are associated with colonization and infection. In addition, their isolation from a normally sterile site is likely to be clinically significant and warrants antimicrobial treatment.

## Introduction

*Escherichia coli* are Gram-negative bacteria belonging to the *Enterobacteriaceae* family, which are ubiquitous colonizers of the gastro-intestinal (GI) tract in humans and companion animals. The *Enterobacteriaceae*, most notably *E. coli* and *Klebsiella pneumoniae*, are among the most important agents of serious hospital acquired and community-onset bacterial infections in humans and resistance to antimicrobial agents in these bacteria has become an increasingly serious problem (Paterson, 2006). Over the last two decades, extended-spectrum β-lactamase (ESBL)-producing *E. coli* have been isolated with increasing frequency from human and animal samples (Coque et al., 2008; Dolejska et al., 2013). ESBLs confer resistance to extended-spectrum cephalosporins (ESCs, e.g., ceftiofur, cefpodoxime, ceftazidime, ceftriaxone) and monobactams, and often express resistance to non-β-lactam antimicrobials leaving only limited therapeutic options (Pitout and Laupland, 2008). In addition, isolates carrying plasmid-mediated cephalosporinases (pAmpC) have a broader spectrum of resistance, including the cephamycins and are not inhibited by β-lactamase inhibitors, resulting in resistance to almost all therapeutically available β-lactam agents (Pitout, 2012). *E. coli* is commonly identified from clinical infections in companion animals, including urinary, respiratory, skin and soft tissues, GI tract, joint and wound infections (Guardabassi et al., 2004; Zogg et al., 2018). In veterinary medicine, the β-lactam agents are arguably the most important and widely used antimicrobial class for treating bacterial infections. Recent United Kingdom data shows that β-lactam agents are prescribed in 43.6% of canine and 70.8% of feline antimicrobial agent prescriptions (Singleton et al., 2017). Their widespread usage may be due to their safety, antimicrobial spectrum, availability of orally bioavailable formulations, favorable pharmacokinetic properties for treating commonly encountered infections and the low cost of many products. First generation cephalosporins and amoxicillin + clavulanic acid are among the most commonly prescribed drugs for dogs (Murphy et al., 2012), whilst penicillin (with or without an aminoglycoside) is commonly prescribed for horses (Hughes et al., 2013). Worryingly, recent data has shown that cefovecin, a third generation cephalosporin, has become the most frequently prescribed antimicrobial agent in the United Kingdom for cats (Singleton et al., 2017).

Similar to the epidemiology of ESBL associated infections in people, the CTX-M type ESBLs (CTX-M-1, -14, and -15) are common amongst companion animal isolates and SHV-12 and TEM-52 have also been reported (Ewers et al., 2012). In addition, there seems to be a match in the geographic distribution of the human and animal CTX-M enzymes, with CTX-M-1 predominating in Africa and Europe, CTX-M-14 in Asia and North America and CTX-M-15 β-lactamase in North America, Europe, and Africa (Rubin and Pitout, 2014).

The occurrence of ESBL/AmpC producing *E. coli* has been reported in both healthy and diseased companion animals (Gandolfi-Decristophoris et al., 2013; Bogaerts et al., 2015), with some reports indicating high fecal carriage rates amongst healthy animals (Maddox et al., 2012; Baede et al., 2015). However, the clinical significance of these isolates and their involvement in animal disease occurring as either opportunistic pathogens or simple colonizers, has been very rarely investigated. Additionally, the lack of clinical and laboratory guidelines for the management of ESBL/pAmpC-producing animal infections, leads to important dilemmas for veterinary microbiologists in deciding when is appropriate to provide susceptibility results to support antimicrobial therapy for these infections. Additional clinical or laboratory information, which could support the distinction between colonization and true infection associated with ESBL/AmpC producing *Enterobacteriaceae* is critical in order to avoid unnecessary use of antimicrobials in companion animals.

Thus, this study aimed to characterize ESBL/pAmpC producing *E. coli* isolates from diseased companion animals (including dogs, cats and horses), with a focus on relating their genetic background to clinical and paraclinical findings in order to determine the role they play in companion animal infections.

## Materials and Methods

### Bacterial Isolates

*Escherichia coli* isolates were obtained from companion animal clinical specimens submitted to the Microbiology Diagnostic Laboratory, Liverpool Institute of Veterinary Science between January 2010 and August 2016. Clinical specimens, mainly originated from animals admitted to the University of Liverpool Small Animal and Equine Referral Hospitals, included urine samples collected by cystocentesis, body fluids, tissue biopsy samples, bile and/or liver biopsy, swabs from skin infections and otitis externa cases, fecal samples from diarrheic patients or animals suspected of GI disease. These were plated out aerobically on culture media following site-specific culture protocols, including 5% sheep blood agar (Oxoid, Basingstoke, United Kingdom) and were incubated at 37°C for 1–7 days. Clinical isolates presumptively identified as *E. coli* based on a positive reaction on Eosin Methylene Blue Agar (EMBA; Oxoid, Basingstoke, United Kingdom) and which showed reduced susceptibility to cefpodoxime (10 μg) and/or cefoxitin, were selected for this study. Species identification of clinical isolates was performed using API 20E identification kits (Biomerieux, France) and also by PCR detection of the *uidA* gene for confirmation of *E. coli* (McDaniels et al., 1996). Bacterial culture data, including specimen site, the type of bacterial growth obtained (pure or mixed, repeat *E. coli* isolation from the same case) and the number of bacterial species isolated from each specimen were recorded in the Veterinary Diagnostic Laboratory database.

### Phenotypic Testing for ESBL/pAmpC Production

Cefpodoxime was used as a marker for extended-spectrum cephalosporin resistance (ESCR) and for ESBL screening. All cefpodoxime-resistant *E. coli* isolates were tested for ESBL production by combination disc testing (CDT) using third generation cephalosporins alone (cefpodoxime, ceftazidime, and cefotaxime) and in combination with clavulanic-acid (MAST Group, United Kingdom) on Mueller-Hinton Agar (Oxoid, Basingstoke, United Kingdom). Results were interpreted according to the Clinical and Laboratory Standards Institute (CLSI) interpretative criteria (CLSI, 2012). In addition, isolates displaying resistance to cefoxitin (30 μg) and cefotaxime (30 μg) or ceftazidime (30 μg), were considered presumptive AmpC producers (CLSI, 2012). To demonstrate the co-production of ESBL and AmpC β-lactamase, cefoxitin resistant isolates were further tested with cefepime (30 μg) and cefepime/clavulanic acid (30/10 μg). *E. coli* ATCC 25922 was used as control strain for susceptibility testing, whilst ESBL and pAmpC producing *E. coli* isolates, previously confirmed by PCR and sequencing, were used as positive controls for the phenotypic ESBL/AmpC testing. Ertapenem (10 μg) and meropenem (10 μg) were also included routinely for carbapenem-resistance screening whilst ciprofloxacin (5 μg) and enrofloxacin (5 μg) were used for confirming fluoroquinolone-resistance in isolates typed to the ST131 H30Rx lineage.

### Characterization of ESBL and pAmpC Resistance Genes

Cell lysates obtained from all investigated isolates were screened by PCR product sequencing for the presence of genes encoding for β-lactamases (*bla*_CTX-M_, *bla*_SHV_, *bla*_TEM_, *bla*_OXA_) and plasmid-mediated *bla*_AmpC_ variants (ACC, FOX, MOX, DHA, CIT, and EBC) as previously described (Essack et al., 2001; Pérez-Pérez and Hanson, 2002; Dallenne et al., 2010). DNA sequencing was performed on amplicons for all the CTX-M and pAmpC positive isolates. For this, PCR amplicons were purified with the Macherey-Nagel NucleoSpin Gel and PCR Clean-up (Thermo Fisher Scientific, United Kingdom). The nucleotide sequences were compared with those present in GenBank^1^ to identify the specific gene variants involved.

### Molecular Typing

An extended PCR scheme described by Clermont et al. (2013) was used to assign the ESBL/AmpC *E. coli* isolates to one of the seven phylogenetic groups (A, B1, B2, C, D, E, F). Isolates not matching any of the profiles of the initial quadruplex scheme were recorded as Unknown phylogroup (U). Selected CTX-M-positive isolates [at least one from each site of infection and carrying unique resistance genes combinations (i.e., *bla*_CTX-M_ and *bla*_TEM_, or *bla*_CTX-M_ and *bla*_SHV_)] were further typed by multi-locus sequence typing (MLST) which was performed as previously described (Wirth et al., 2006). All isolates which typed to phylogroup B2 were screened for the presence of the O25b-ST131 clone by allele-specific PCR (Clermont et al., 2008). Identification of *E. coli* ST131 clade, including the subclade C/H30, C/H30-R with its H30Rx and non-Rx sublineages was performed by PCR-based multiplex ST131 clade assay (Matsumura et al., 2017).

### Virulence Gene and Extraintestinal Pathogenic *E. coli* (ExPEC) Characterization

All ESCR isolates were screened by a multiplex PCR assay designed to detect virulence markers likely to be associated with extraintestinal pathogenic *E. coli* (ExPEC) (Johnson et al., 2015). ExPEC status was assigned according to the definition proposed by Johnson and Russo (2005) which defined ExPEC isolates on the basis of the presence of ≥2 virulence associated genes (VAGs) or gene sets, including *papAH* and/or *papC, sfa/focDE* [central region of *sfa* (S fimbriae) and *foc* (F1C fimbriae) operons], *afa/draBC* (Dr antigen-binding adhesins), *kpsMII* (group II capsule Polysaccharides*)*, and *iutA* (ferric aerobactin receptor) (Johnson et al., 2015). A virulence score was assigned to each isolate based on the number of virulence genes detected, with *pap* elements counting collectively as a single trait.

### Clinical and Paraclinical Data

To evaluate the likelihood of ESCR *E. coli* isolates being associated with clinical infections, overall bacterial culture results (including results of repeated sampling from the same case), hematological results (where available) and clinical data were collated. From these data, and after reviewing referral letters, patient’s clinical finding and ancillary tests, which were available in 146/164 cases, a decision of evidence of infection was made for each case. Confirmation of association with infection was provided by the presence of at least one of the following: rods/coccobacilli in cytology smears (either Diff-quick or Gram-stained), raised peripheral white blood cell count, raised inflammatory markers (C-reactive protein when tested) and/or the final clinical diagnosis of infection. Where none of these criteria were met and the isolate was obtained from a mixed bacterial culture, the isolation of an ESBL/AmpC-producing *E. coli* was considered to be due to contamination.

### Statistical Analysis

GraphPad Prism 7.0 (GraphPad Software, Inc., San Diego, CA, United States) was used for data analysis. We used the chi-squared test to assess the correlation between ExPEC status, phylogroup and presence of antimicrobial resistance genes. Mann–Whitney *U* test was used to assess the virulence factor score correlation with phylogroup and presence of antimicrobial resistance genes. *P*-value < 0.05 was considered statistically significant.

## Results

### Prevalence of ESCR, ESBL, and pAmpC Producing *E. coli* Isolates

During the study period (January 2010–August 2016), 876 *E. coli* isolates (dogs *n* = 748, cats *n* = 76 and horses *n* = 52) were obtained from the clinical specimens submitted from companion animals. Of these, ESCR was identified in 164 (18.7%) non-repetitive *E. coli* isolates obtained from dogs (*n* = 138), cats (*n* = 8) and horses (*n* = 18) which displayed inhibition zones (≤25 mm) for cefpodoxime (CPD) with or without cefoxitin resistance.

The ESCR *E. coli* isolates were cultured from urine (*n* = 49), wound swabs (*n* = 41), GI tract samples, mainly feces from diarrheic animals (*n* = 30), skin infections or otitis cases (*n* = 23), body fluids or tissue biopsies (*n* = 9), bile or liver samples (*n* = 12). Combination disc tests for phenotypic ESBL testing of the 164 ESCR isolates resulted in 53 isolates displaying an ESBL phenotype (53/164, 32.3%), 67 isolates showed a presumptive AmpC phenotype (67/164, 40.9%) and 11 isolates (11/164, 6.7%) demonstrated a phenotype suggesting mixed ESBL/AmpC co-production (resistance to cefoxitin, cefotaxime or ceftazidime and demonstrating synergy when tested with cefepime, a fourth generation cephalosporin and cefepime/clavulanic acid). Thirty-three ESCR isolates (33/164, 20.0%) produced inconclusive phenotypic ESBL/pAmpC results (Table 1). No carbapenem-resistance was detected in the ESCR isolates.

**Table 1:** Phenotypic and genotypic characterization of extended-spectrum cephalosporin resistant *Escherichia coli* isolates obtained from companion animals clinical specimens.

Phenotype	β-lactamases enzyme	Number of isolates	Phylogroup	ST	Year of isolation	Source	Body site
ESBL (*n* = 53)	CTX-M-15	7	A, B1 (*n* = 4) B2 (*n* = 2) U (*n* = 1)	86 (1) 23 (1) 623 (1) 3880 (1) 4184 (1)	2010 (*n* = 1), 2013 (*n* = 1), 2014 (*n* = 3)	Dog (*n* = 5), Horse (*n* = 2)	GI tract (*n* = 3), SI/O (*n* = 1), Urine (*n* = 1), Wound (*n* = 2)
	CTX-M-15, OXA	10	A, B1 (*n* = 2) B2 (*n* = 3) C (*n* = 4) D (*n* = 1)	131 (3) 167 (1) 1284 (1) 2348 (1)	2010 (*n* = 4), 2011 (*n* = 3), 2012 (*n* = 1)	Dog (*n* = 9), Horse (*n* = 1)	GI tract (*n* = 4), Hepato-biliary (*n* = 1), Urine (*n* = 1), SI/O (*n* = 3), Wound (*n* = 1)
	CTX-M-15, TEM, OXA	2	D (*n* = 1) F (*n* = 1)	648 (1)	2011	Dog	BF/BP (*n* = 2), SI/O (*n* = 3)
	CTX-M-15, TEM	3	A	10 (1) 167 (1)	2011	Dog	Wound (*n* = 3)
	CTX-M-14	6	A (*n* = 3) B2 (*n* = 2) C (*n* = 1)	10 (2) 95 (1) 209 (1)	2012 (*n* = 2) 2014 (*n* = 1), 2015 (*n* = 3)	Dog (*n* = 2), Cat (*n* = 1), Horse (*n* = 1)	GI tract (*n* = 1), SI/O (*n* = 1), Urine (*n* = 4)
	CTX-M-14, TEM	8	A (*n* = 7), D (*n* = 1)	10 (1) 209 (1) 617 (2)	2010 (*n* = 5), 2015 (*n* = 2), 2016 (*n* = 1)	Dog (*n* = 8)	BF/BP (*n* = 2), Hepato-biliary (*n* = 2) GI tract (*n* = 1), Urine (*n* = 3)
	CTX-M-55	2	B2 (*n* = 2)	1340 (2)	2015 (*n* = 2)	Dog (*n* = 2)	GI tract (*n* = 2)
	CTX-M-55, TEM	3	A (*n* = 1) B2 (*n* = 2)	167 (1) 1340 (2)	2015 (*n* = 2), 2016 (*n* = 1)	Dog (*n* = 3)	Urine (*n* = 2), GI tract (*n* = 1),
	CTX-M-55, OXA	1	B2	131	2015	Dog	Urine
	CTX-M-55, TEM, SHV-12	1	B1	5036	2013	Horse	Wound
	CTX-M-1	3	B1 (*n* = 2) D	47923509	2013 (*n* = 2) 2014 (*n* = 1)	Dog	GI tract
	CTX-M-1, TEM	1	B1	641	2013	Horse	Wound
	CTX-M-27	1	B2	131	2010	Dog	Hepato-biliary
	CTX-M-28, TEM	3	B1 (*n* = 3)	3743448 (2)	2015	Dog (*n* = 2), Horse (*n* = 1)	SI/O (*n* = 2), Wound (*n* = 1)
	CTX-M-24, TEM	1	A	–	2014	Dog	Wound (*n* = 1)
	CTX-M-24, OXA	1	C	3163	2012	Dog	Wound (*n* = 1)
AmpC (*n* = 67)	CMY-2	23	B1 (*n* = 4) B2 (*n* = 9) C/F (*n* = 3) D (*n* = 7)	–	2010 (*n* = 2), 2011 (*n* = 1), 2012 (*n* = 6) 2013 (*n* = 4), 2014 (*n* = 5), 2015 (*n* = 4), 2016 (*n* = 1)	Dog (*n* = 23)	Wound (*n* = 7), Urine (*n* = 6), GI tract (*n* = 5), SI/O (*n* = 4), Hepato-biliary (*n* = 1)
	CMY-2, TEM	30	A/B1 (*n* = 12) B2 (*n* = 12) C (*n* = 3) F (*n* = 3)	2348 (1)	2010 (*n* = 1), 2011 (*n* = 2), 2012 (*n* = 12) 2013 (*n* = 4), 2014 (*n* = 4), 2015 (*n* = 6)	Dog (*n* = 28), Cat (*n* = 1), Horse (*n* = 1)	Urine (*n* = 15), GI tract (*n* = 5), Wound (*n* = 4), BF/BP (*n* = 3), Hepato-biliary (*n* = 2), SI/O (*n* = 1)
	CMY-2, TEM, OXA	3	B2 (*n* = 1) C (*n* = 1) D (*n* = 1)	410 (1)	2012 (*n* = 1), 2013 (*n* = 2)	Dog	Urine (*n* = 1), SI/O (*n* = 1), GI tract (*n* = 1)
	CMY-2, OXA	2	A (*n* = 1) C (*n* = 1)	–	2010 (*n* = 1), 2012 (*n* = 1)	Dog (*n* = 2)	Wound (*n* = 2)
	CMY-2, TEM, DHA	1	B2	–	2015	Dog	SI/O
	DHA	1	B1	–	2015	Dog	Wound
	DHA, TEM	3	A/B1 (*n* = 2) F (*n* = 1)	–	2013 (*n* = 1), 2015 (*n* = 1), 2016 (*n* = 1)	Dog	Hepato-biliary (*n* = 1), Urine (*n* = 1), Wound (*n* = 1)
	DHA, TEM, OXA	1	B1	–	2015	Dog	Wound
	EBC	1	B2	–	2014	Horse	Wound
	EBC, TEM	2	B2	–	2014	Horse	Wound (*n* = 2)
ESBL/AmpC(*n* = 11)	CTX-M-15, OXA, CMY-2	4	B2 (*n* = 1) C (*n* = 3)	1485 (1)	2012 (*n* = 3), 2013 (*n* = 1)	Dog	BF/BP (*n* = 1), SI/O (*n* = 1), Urine (*n* = 1), Wound (*n* = 1)
	CTX-M-15, TEM, OXA, CMY-2	3	B2	410 (1) 3163 (1)	2011 (*n* = 1), 2012 (*n* = 2)	Dog	GI tract
	CTX-M-15, TEM, OXA, DHA	1	D	–	2013	Dog	SI/O
	CTX-M-24, CMY-2, TEM, OXA	1	U	–	2013	Dog	Hepato-biliary
	CTX-M-24, CMY-2, TEM, SHV-12	1	C	410	2013	Horse	BF/BP
	CTX-M-55, TEM, CMY- 2	1	A	167	2013	Dog	GI tract (*n* = 1)
Inconclusive phenotype (*n* = 33)	TEM, OXA	1	B1	–	2014	Dog	Bile
	TEM	13	A/B1 (*n* = 10) B2 (*n* = 2) U (*n* = 1)	–	2010 (*n* = 2), 2011 (*n* = 1), 2012 (*n* = 1), 2013 (*n* = 1), 2014 (*n* = 3), 2015 (*n* = 2), 2016 (*n* = 3)	Dog (*n* = 8), Cat (*n* = 2), Horse (*n* = 3)	BF/BP (*n* = 1), GI tract (*n* = 2), SI/O (*n* = 2), Urine (*n* = 5), Wound (*n* = 3)
	OXA	4	B1 (*n* = 1), F (*n* = 2), U (*n* = 1)	–	2012 (*n* = 3), 2014 (*n* = 1)	Dog (*n* = 3), Horse (*n* = 1)	Wound (*n* = 2), Urine (*n* = 1), SI/O (*n* = 1)
	–	15	B1 (*n* = 2) B2 (*n* = 12) C (*n* = 1)	–	2010 (*n* = 1), 2011 (*n* = 1), 2012 (*n* = 1), 2013 (*n* = 2), 2014 (*n* = 3), 2015 (*n* = 5), 2016 (*n* = 2)	Dog (*n* = 11), Cat (*n* = 4)	Urine (*n* = 7), Wound (*n* = 3), SI/O (*n* = 3) Hepato-biliary (*n* = 2)


*ST, sequence type by multi-locus sequence typing; BF/BP, body fluids/biopsies; SI/O, skin infection/otitis.*

### Molecular Characterization of ESBL/pAmpC Genes

Overall, ESBL and/or pAmpC encoding genes were found in 79.9% (131/164) of the ESCR *E. coli* isolates. *bla*_CTX-M_ genes were the most common ESBL genotype (48.9%, 64/131) of which the most prevalent were *bla*_CTX-M-15_ (46.9%, 30/64), *bla*_CTX-M-14_ (21.9%, 14/64), and *bla*_CTX-M-55_ (12.5%, 8/64). *bla*_CTX-M-1_ and *bla*_CTX-M-24_ were each found in four isolates; *bla*_CTX-M-28_ was found in three isolates whilst only one *bla*_CTX-M-27_ *E. coli* isolate was identified in a liver tissue biopsy from a dog. In addition, *bla*_SHV -12_ (*n* = 2) was detected in two isolates, which both co-harbored CTX-M genes (*bla*_CTX-M-55_ and *bla*_CTX-M-24,_ respectively). The vast majority of CTX-M-type ESBL producing isolates also carried *bla*_TEM_ (*n* = 29/64) and/or *bla*_OXA_ (*n* = 23/64). A number of ESCR isolates (*n* = 18/164) only produced TEM or OXA β-lactamases whilst in 15/164 isolates, most of which belonged to phylogroup B2 (*n* = 12/15), none of the investigated *bla* genes were detected. Resistance to ESC via plasmid mediated AmpC genes was detected in 51.2% (67/131) of the ESBL and/or pAmpC producing isolates, where *bla*_CMY -2_ was the most common pAmpC gene identified (59/67), whilst in some isolates pAmpC genes were co-harbored with *bla*_CTX-M-15_ (*n* = 8), *bla*_CTX-M-24_ (*n* = 2) or *bla*_CTX-M-55_ (*n* = 1). Additionally, *bla*_DHA_ and *bla*_EBC_ were detected in seven canine isolates and three equine isolates, respectively. No *bla*_ACC_, *bla*_MOX_ or *bla*_FOX_ plasmid mediated AmpC resistance genes were detected in the investigated isolates.

### Molecular Typing of *E. coli* Isolates

Phylogenetic group B2, which is associated with extra-intestinal infections in humans, was the most commonly identified phylogroup among the ESCR *E. coli* isolates (33.5%, 55/164); phylogroups D and F, which are closely related, and can also be associated with extra-intestinal infections, represented 12.20% (20/164) of the isolates. The phylogroups generally regarded as commensal strains (A, B1) were also associated with ESCR where phylogroup B1 was the most predominant (*n* = 35, 21.3%), closely followed by phylogroup A (*n* = 31, 18.9%), C (*n* = 19, 11.5%), and U (*n* = 4, 2.4%).

MLST typing identified ST410, recently reported as a new successful pandemic clone (Schønning et al., 2018), as the most prevalent amongst ESBL/pAmpC producing and MLST tested isolates (6/131, all phylogroup C), three of which were both CTX-M- and pAmpC –producers. ST410 was identified in isolates obtained from five dog clinical specimens [urine (*n* = 2), abdominal fluid (*n* = 2), tissue biopsy] and one from a horse abdominal fluid isolate. In addition, other important human *E. coli* clones involved in ESBL dissemination were identified amongst CTX-M-producers such as ST10 in four dog isolates, three CTX-M-14 and one CTX-M-15 producers, all phylogroup A, obtained from wound, urine (*n* = 2) and tissue biopsy; ST167 in one CTX-M-15 and two CTX-M-55 dog isolates from wound, feces and urine, and also one equine CTX-M-15 producing horse isolate obtained from a wound swab; ST648 (phylogroup D) was identified in one isolate obtained from an interdigital abscess from a dog.

Five *E. coli* isolates were typed to ST131-B2 (5/164, 3.1%) by both allele specific PCR and MLST and were CTX-M-15, -14, -55, or -27 producers. All ST131-B2 isolates were from dogs with three isolates from sterile sites (two from bile, one from urine) whilst two isolates were from a colon biopsy and a skin swab. ST131-clade specific PCR showed that 4/5 ST131 isolates belonged to Clade C/H30; by using the same assay, one isolate carrying *bla*_CTX-M-27_ was typed to the recently described sub-clade C1-M27; three isolates carrying *bla*_CTX-M-15_ and showing fluoroquinolone resistance were typed to the subclade C2 known as H30Rx (C2/H30Rx). The remaining ST131 isolates carried *bla*_CTX-M-55_ and demonstrated fluoroquinolone resistance but remained unclassified by this assay.

### Virulence Score (VS) and ExPEC Status of ESCR Isolates

The majority of ESCR *E. coli* isolates included in this study were obtained from non-GI tract sites (103/131, 81.7%). As expected, most of the isolates with virulence factor (VF) scores >2 belonged to phylogroup B2 (32/55 isolates, 58%) and phylogroup D and F (in 5/20 isolates, 25%), followed by phylogroup A (5/31 isolates 16%), phylogroup C 19 isolates (2/19 isolates 10.5%) and phylogroup B1 (3/35 isolates8.6%).

P fimbrial structural subunits encoded by *papA, papC* or *papE* (75/164, 45.7%) were the most commonly identified virulence genes, followed by *iutA* (61/164, 37.2%), which encodes for a ferric aerobactin receptor involved in the iron uptake and *kps*II gene (42/164, 25.6%) which is involved in capsular polysaccharide production. The B2 phylogroup showed a significantly higher virulence score compared to all the other groups (*p* < 0.01) and more than half of B2 strains (32/55) where defined as ExPEC. Phylogroups D and F, which can also be associated with ExPEC, demonstrated a higher virulence score compared to isolates belonging to group A, B1, C, and U but this difference was not statistically significant (Table 2).

**Table 2:** Distribution of average virulence scores and ExPEC status in relation to their phylogroups and resistance genes identified.

	Phylogroup *n (%)*				ESBL/AmpC type *n (%)*					
		
	A/B1 (*n* = 66)	B2 (*n* = 55)	D and F (*n* = 20)	C and U (*n* = 23)	CTX-M group 1 (*n* = 36)	CTX-M- group 9 (*n* = 18)	AmpC (*n* = 67)	ESBL/AmpC (*n* = 11)	Additional β-lactamase producers (*n* = 18)	Inconclusive phenotype^∗^ (*n* = 15)
**Average virulence**										
**score**	0.76	2.70	1.00	0.91	1.11	1.82	1.28	0.7	1.47	3.4
*hlyD*	1	14	1	0	2	1	6	0	2	5
*papA*	4	17	1	2	3	6	8	0	2	5
*kpsII*	3	29	8	2	9	2	20	1	4	6
*iutA*	26	18	6	11	17	9	20	6	8	1
*papC*	6	16	0	3	2	6	7	0	3	7
*sfa1*	3	16	1	1	1	1	8	0	3	8
*afa1*	0	2	0	0	2	0	0	0	0	0
*papE*	6	18	2	2	2	6	6	0	5	9
*cnf1*	3	17	0	0	3	1	6	0	1	9
**ExPEC**	8 (12.12%)	32 (58.18%)	5 (25.00%)	3 (13.05%)	8 (22.22%)	8 (44.44%)	13 (19.40%)	0 (0%)	6 (33.33%)	8 (53.33%)


*ExPEC, extraintestinal pathogenic *E. coli*, defined operationally as the presence of >2 of the virulence genes investigated (papA and/or papC, counted as one). ^∗^Isolates lacked all beta-lactam resistance genes, which they were screened for.*

Analysis of virulence factor scores, in relation to carriage of resistance genes, also showed differences in the frequency of virulence genes amongst isolates carrying or lacking ESBL/pAmpC genes. In particular, the ExPEC status and virulence score average were higher for isolates lacking the screened ESBL/pAmpC resistance genes (here defined as isolates with an inconclusive phenotype) and lower for isolates possessing both ESBL and AmpC genes (*p* < 0.05) (Table 2).

### Body Site Distribution

**FIGURE 1:**
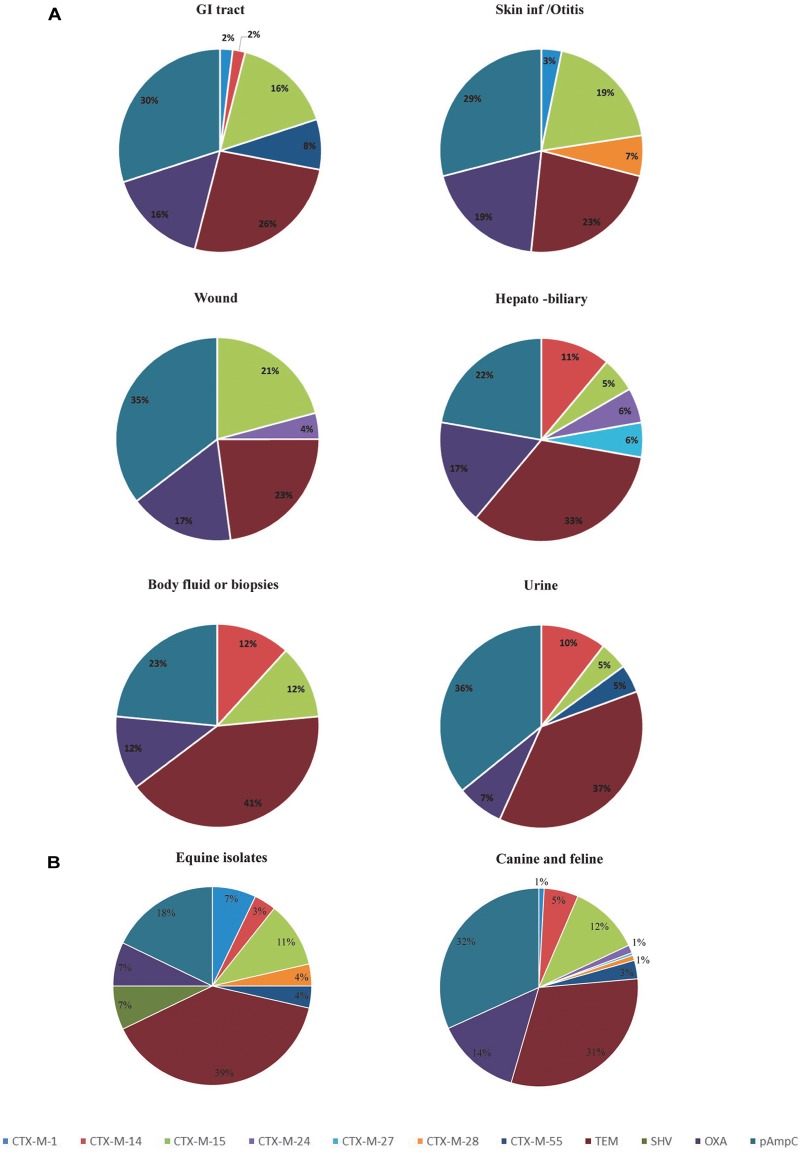
Diversity and distribution of ESBL and pAmpC producing *Escherichia coli* according to the clinical specimens **(A)** and species **(B)**. GI, Gastrointestinal tract.

Analysis of the frequency of ESBL/pAmpC genotypes according to the site of isolation, shows that isolates from the GI tract, hepatobiliary infections and urine carry a greater variety of ESBL/pAmpC resistance genes compared to isolates from the skin/soft tissue infections, body fluids and wound infections (Figure 1A). With regard to the host species involved, horse isolates showed a wider variety of *bla* genes despite the low numbers of isolates compared to canine isolates (Figure 1B). However, *bla*_CTX-M-15_ was the predominant ESBL genotype identified in both canine and equine isolates (19.9 and 16.6% of isolates, respectively), as well as in GI tract (35.7%), skin/otitis isolates (31.6%) and wound isolates (19.2%) from cats and dogs. CTX-M-14 β-lactamase producers, although rare in wound infections and GI tract isolates (0 and 2%, respectively), were a relatively common ESBL type found in isolates from body fluids/biopsy samples (25.0%), hepatobiliary infections (18.2%), and urine (15.9%). In addition, around half of the ESCR isolates obtained from dogs harbored pAmpC genes (72/138, 52.2%), either alone or in combination with other β-lactamase encoding genes, with frequencies ranging from 36% for hepatobiliary infections to 55% in the case of urine samples. Moreover, *bla*_CMY -2_ alone or in combination with *bla*_TEM_ was the most common genotype associated with ESCR *E. coli* isolates from UTI from dogs (23/46, 50.0%).

### Clinical/Paraclinical Data Analysis

Clinical or laboratory data used to evaluate evidence of infection was available in 111 of 146 cases (76.0%). Overall, 70 ESCR *E. coli* isolates were obtained from normally sterile body sites (body fluids/biopsy, urine collected by cystocentesis and hepatobiliary samples) and 54 of these were pure cultures (54/70, 77.1%). Whereas from 94 ESCR isolates, obtained from body sites populated with commensal flora (skin, ear canal, GI tract), only 14 *E. coli* isolates were obtained in pure growth (14/94, 14.8%) (*p* < 0.0001).

Bacterial culture results of ESCR *E. coli* isolates (Table 3), also shows that the vast majority of the isolates originating from urinary tract infections (UTIs) were obtained in pure culture (45/49, 91.8%) and in almost half of the cases, the clinical records showed repeated isolation of an ESBL/AmpC producing isolate (20/49, 40.8%) indicating persistence or re-infection; this was significantly higher than the frequency of repeated isolation from skin/ear infections (*p* = 0.0029) and GI tract (*p* < 0.00001). In addition, in the majority of UTI cases where an ESCR *E. coli* isolate was obtained (30/49, 61.2%), there was at least one clinical or paraclinical test result supportive of infection (either rods on cytology, hematuria or pollakiuria, neutrophilia). However, in 11 UTI cases (11/49, 22.4%) isolation of ESBL/pAmpC producing bacteria was not accompanied by clinical signs, suggesting transient colonization of the bladder or urinary tract or possibly subclinical infection; clinical/paraclinical data was missing in 8 UTI cases. In contrast, the majority of ESCR isolates from skin infections/otitis, GI tract and wounds were obtained in mixed cultures (80/94, 85.1%). The role played by the ESCR or specifically the ESBL/AmpC producing bacteria is difficult to ascertain in samples yielding a mixed bacterial growth (polymicrobial cultures often with other Gram-negative bacteria also involved); however, in most of these cases (64/94, 69.1%) there was clinical evidence of infection (mainly inflammation and purulent discharge).

**Table 3:** Clinical and laboratory data associated with extended-spectrum cephalosporin resistant *Escherichia coli* clinical isolates.

	Phylogroup(s) (*n*)	VF		ST (*n*- if >1 isolates)	ESBL/AmpCBeta-lactamase	Narrow-spectrum beta-lactamase	Type of growth		Repeat isolation		Evidence of clinical infection	
								
		≥2(*n*)	≤2(*n*)				Pure (*n*)	Mixed (*n*)	Yes (*n*)	No (*n*)	Yes (*n*)	No (*n*)
Urine (*n* = 49)	B2 (16)		1		CTX-M-14		15	1	3	12	10	5
		1	1	ST1340; ST131	CTX-M-55	TEM (1)OXA (1)						
		5	1		CMY-2	TEM (4)						
		2				TEM						
		5				–						
	D and F (6)		1		DHA	TEM	6	0	3	2	4	0
			4		CMY-2	TEM (1)						
		1			CTX-M-14	TEM						
	A (6)		1	ST167;	CTX-M-55		5	1	4	2	5	1
		4	1	ST10 (2); ST209 (2); ST617	CTX-M-14	TEM (3)						
	B1 (15)		1	ST23	CTX-M-15		14	1	8	7	7	4
		1	8		CMY-2	TEM (8)						
		1	2			TEM (2),OXA (1)						
			2									
	C (5)		1		CTX-M-15, CMY-2	OXA	4	1	2	3	4	1
			1	ST410	CTX-M-15	OXA						
		1	2	ST410 (1)	CMY-2	TEM (3),OXA (1)						
	U		1			TEM	1	0	–	–	–	–
**Total**		**22**	**27**				**45**	**4**	**20**	**26**	**30**	**11**
Body fluid/biopsies (*n* = 9)	B2 (1)		1		CMY-2	TEM	0	1	1	0	1	0
	F (2)		1		CTX-M-15	TEM, OXA	1	1	0	2	1	0
		1		ST2348	CMY-2	TEM						
	A (4)		1			TEM	1	3	2	1	3	0
		1	1	ST10, ST617	CTX-M-14	TEM (2)						
			1		CMY-2	TEM						
	C (2)		1	ST410	CTX-M-15, CMY-2	OXA	2	0	1	1	2	0
			1	ST410	CMY-2, SHV	TEM						
**Total**		**2**	**7**				**4**	**5**	**4**	**4**	**7**	**0**
Hepato-biliary (*n* = 12)	B2 (4)		1	ST131	CTX-M-27		3	1	1	3	4	0
		1		ST131	CTX-M-15	OXA						
		1			CMY-2							
		1										
	F (1)		1		CMY-2	TEM	1	0	0	1	0	1
	A (3)		2		CTX-M-14	TEM (2)	0	3	0	3	3	0
			1		DHA	TEM						
	B1 (1)		1			TEM, OXA	0	1	1	0	1	0
	C (2)		1		CMY-2	TEM	1	1	1	1	1	1
			1									
	U (1)		1		CTX-M-24, CMY-2	TEM, OXA	0	1	1	0	1	0
**Total**		**3**	**9**				**5**	**7**	**4**	**8**	**10**	**2**
Skin infection/otitis (*n* = 23)	B2 (11)	2		ST131, ST3880	CTX-M-15	OXA (1)	1	10	1	9	10	0
			1	ST1485	CTX-M-15, CMY-2	OXA						
		1		ST95	CTX-M-14							
		2	2		CMY-2	TEM						
		3										
	D and F (3)	1	1	ST2348	CTX-M-15	TEM (1), OXA (2)	0	3	0	2	2	0
			1		CMY-2	TEM, OXA						
	A (2)		2			TEM (2)	0	2	1	0	1	0
	B1 (5)		1	ST4792	CTX-M-1		0	5	0	4	3	1
			2	ST448 (2)	CTX-M-28	TEM (2)						
			2		CMY-2	TEM (1)						
	C (1)		1		CTX-M-15	OXA	0	1	1	0	1	0
	U (1)	1				OXA	0	1	0	1	1	0
**Total**		**9**	**14**				**1**	**22**	**3**	**16**	**18**	**1**
GI tract (*n* = 30)	B2 (15)	1	1	ST131; ST623	CTX-M-15	OXA (1)	3	12	3	12	8	7
			2	ST3163 (2)	CTX-M-15, CMY-2	TEM (2), OXA (2)						
		2	1	ST1340 (2)	CTX-M-55	TEM (1)						
		2	6		CMY-2	TEM (5), OXA (1)						
	D (1)		1	ST3509	CTX-M-1		05	1	0	1	0	1
	A (6)		1		CMY-2	TEM	0	5	0	5	1	4
			2	ST1284; ST4184	CTX-M-15	OXA (1)						
			1	ST617	CTX-M-14	TEM						
			1	ST167	CTX-M-55, CMY-2	TEM						
			1			TEM						
	B1 (3)		2		CMY-2		1	2	0	2	2	0
			1			TEM						
	C (4)		1	ST410	CTX-M-15, CMY-2	TEM, OXA	0	4	0	4	2	2
			2		CTX-M-15	OXA (2)						
			1		CTX-M-14							
	U (1)		1		CTX-M-15		0	1	0	1	1	0
**Total**		**5**	**25**				**4**	**25**	**3**	**25**	**14**	**14**
Wound (*n* = 41)	B2 (8)	1	1		CMY-2	TEM (2)	0	8	1	7	7	1
			3		EBC	TEM (2)						
		2	1									
	D and F (7)	2				OXA	1	6	1	6	6	1
			5		CMY-2							
	A (10)		1		CTX-M-24	TEM	3	7	3	7	7	3
			2		CTX-M-15, DHA or CMY	TEM, OXA						
			3	ST167, ST10	CTX-M-15	OXA (1), TEM (2)						
			2		CMY-2	OXA (1), TEM (1)						
			2			TEM						
	C (5)		1	ST410	CTX-M-15, CMY-2	OXA	1	4	2	3	4	1
		1		ST3163	CTX-M 24	OXA						
			3		CMY-2	OXA (1)						
	B1 (11)	1	1	ST641	CTX-M-1	TEM (1)	4	7	2	6	6	2
			2	ST86	CTX-M-15							
			1	ST3743	CTX-M-28	TEM						
			1	ST5036	CTX-M-55, SHV	TEM						
			3		DHA	OXA (1), TEM (2)						
			1		CMY-2	TEM						
			1									
**Total**		**7**	**34**				**9**	**32**	**9**	**29**	**30**	**8**


*ST, sequence type by multi-locus sequence typing; VF, virulence factor score.*

Analysis of *E. coli* phylogroups in relation to the infection site and carriage of resistance genes, shows that the phylogroups usually associated with ExPEC (B2, D, and F) and those regarded as commensals (A, B1, C), were both associated with ESCR and ESBL/AmpC production and were isolated in largely similar proportions from UTIs (22 versus 27), hepatobiliary infections (5 versus 7), GI tract specimens (16 versus 14); however, higher numbers of the ExPEC associated phylogroups (B2, D, and F) were found amongst skin infections and otitis isolates (14 versus 9) and lower numbers amongst body fluids/biopsy isolates (3 versus 6) and wound infections (15 versus 26) (Table 3). Analysis of VF distribution among these two groups [likely ExPEC (B2, D, and F) versus likely commensal (A, B1, and C)] shows that, with the exception of UTI isolates, where there were a similar number of isolates from the two groups with a VF ≥ 2, in general isolates of phylogroups B2, D, and F carried more virulence genes (≥2); this was observed especially in hepatobiliary, skin infections and GI tract isolates. In addition, clinical data analysis also showed that in 70.6% of the infections associated with ESCR *E. coli* of phylogroup B2, D, and F there was clinical/paraclinical evidence of an active infection.

## Discussion

Despite the increasing prevalence of ESBL/pAmpC producing *Enterobacteriaceae* reported in animals and the increasing rate of isolation by veterinary laboratories, there is still little information regarding the role that ESBL/AmpC producing *E. coli* isolates play as infectious agents in diseased companion animals. Many studies have investigated the zoonotic risks and the prevalence of fecal carriage or have characterized the ESBL/AmpC producing isolates from clinical specimens in animals (Dierikx et al., 2012; Hordijk et al., 2013; Maeyama et al., 2018; Zogg et al., 2018). However, very few studies (mainly human) have linked the molecular characteristics of ESBL/pAmpC producing isolates with clinical data in order to determine the clinical significance of ESCR *E. coli* (Sidjabat et al., 2009; Schoevaerdts et al., 2011; Rodríguez-Baño et al., 2012). Thus, the current study investigated the molecular characteristics of ESBL/pAmpC producing *E. coli* isolates from various companion animal infections in relation to clinical and paraclinical information, which could provide an indication of their role as infecting agents or occasional contaminants in clinical specimens. Comparison with our previous work shows an important increase of ESCR *E. coli* prevalence in clinical isolates investigated in our laboratory over time, with a rise from 7% (2010/2011) (Timofte et al., 2016) to 21% in the current study (samples from 2010/2016), highlighting the need for greater understanding of their association with infection in order to improve disease management and infection control.

Molecular characterization has shown that ST410 was the most common ST identified in clinical isolates from dogs and horses in this study, both in CTX-M- and pAmpC producers which is concerning given that this clone was recently described as a high-risk multi-drug resistant (MDR) clone with increased potential for inter-species transmission (Schaufler et al., 2016). Also, recently attention has been drawn to the emergence of *E. coli* D-ST648 in companion animals, a clone which combines characteristics of multidrug resistance and virulence, similar to those observed in *E. coli* ST131-B2 (Ewers et al., 2014). In our study, we describe the isolation of a D-ST648-CTX-M-15 *E. coli* from an interdigital abscess from a dog, and to the best of our knowledge, this has not been reported previously in United Kingdom animals. The pandemic virulent *E. coli* ST131-B2 clone was rare among the ESCR isolates analyzed (five isolates); nonetheless, its isolation occurred in three different years (2010, 2011, and 2015) which suggests persistence in animal populations and raises the potential of zoonotic risks represented by ESBL-producing *E. coli* in companion animals. Previous studies have shown that ST131-B2 has driven the dissemination of the CTX-M β-lactamase (particularly CTX-M-15) worldwide (Petty et al., 2014) and recent epidemiologic and whole genome phylogeny studies have shown that the clonal structure of *E. coli* ST131 is highly variable including subclones with particular resistance traits (Price et al., 2013). These studies have also shown that specific sublineages are particularly responsible for ST131/CTX-M-15 dissemination, especially those recently named as clone C or C/H30, which encompasses two sublineages [C/H30-R (non-Rx) and C2/H30Rx] both characterized by fluoroquinolone resistance (Matsumura et al., 2017). In our study, one *bla*_CTX-M-27_ positive isolate was typed to the C1/H30-R (recently described as the C1-M27 sublineage) and three *bla*_CTX-M-15_ and fluoroquinolone resistant isolates were typed to the C2/H30Rx sublineage. The C1-M27 clone is responsible for the epidemic spread of ST131-CTX-M-27 in Japan (Matsumura et al., 2015) where a high prevalence of *E. coli* ST131 H30R/non-Rx lineage (likely the C1-M27 sublineage) was also found in isolates from dogs (Maeyama et al., 2018). A recent study looking at the prevalence and characteristics of ST131 clone among ESBL-producing *E. coli* colonized patients from four European hospitals, also showed a changing epidemiology of ST131 in Europe and the emergence of C1-M27 clade (Merino et al., 2018). Ghosh et al. (2017) examined the presence of ST131-CTX-M-27 producing *E. coli* amongst isolates from the environment, food, livestock, humans, and companion animals in Germany and found that this lineage was exclusively found in human isolates and showed an increasing prevalence from 0 to 45% over a period of 7 years. *E. coli* ST131 H30/H30-Rx subclones causing UTI in companion animals in Europe have been previously reported in companion animals from Portugal (Belas et al., 2018) but to the best of our knowledge, this is the first report of ST131 C1-M27 sublineage in companion animals outside Japan.

Overall, the molecular characterization of resistance genes and genetic background of the isolates analyzed this study has identified a diversity of *bla*_CTX-M_ genes in *E. coli* isolates. The *bla*_CTX-M_ variants, CTX-M-14 and CTX-M-15 generally predominate amongst human and animal *E. coli* isolates; however, our study shows a decreasing occurrence of CTX-M-15 β-lactamase producers over the last few years in favor of new variants, in particular CTX-M-55. In the United Kingdom, this latter *bla*_CTX-M_ variant has been isolated from turkey meat (Randall et al., 2014; Parker et al., 2016), but it was not previously identified from companion animals in the United Kingdom or Europe. However, CTX-M-55 is among the predominant gene type in *E. coli* isolated from companion animals in Asian countries (Kawamura et al., 2017).

Phylogroup typing of ESCR *E. coli* isolates showed a high occurrence of B2 strains (33.5%) which is markedly higher than the frequency reported in a similar study conducted in Germany where 2.4% of the strains were assigned to this phylogroup (Schmiedel et al., 2014; Wagner et al., 2014). B2 strains are frequently associated with a higher number of virulence determinants (Valentin et al., 2014) and as expected, the virulence score of B2 strains (and to a lesser extent D and F) in this study was markedly higher than of other phylogroups; therefore, isolates typed to phylogroup B2, D, or F were more frequently classified as ExPEC. Furthermore, in the majority of the infections associated with phylogroups B2, D, and F there was clinical/paraclinical evidence of infection that supports their association with true infection. The average virulence score in the ESCR isolates was lower in isolates carrying both ESBL/pAmpC genes (VS = 0.7) and higher in isolates lacking these genes and categorized as inconclusive phenotype (VS = 3.4). This is in accordance with the general concept that in most cases, increased resistance in *E. coli* isolates can be associated with decreased virulence due to survival fitness costs (Beceiro et al., 2013). However, the existence of already established (ST131) or emerging (ST648) clones combining virulence and multidrug-resistance via production of CTX-M β-lactamase (Ewers et al., 2014; Petty et al., 2014), provides the evidence that the opposite is also true and occurrence of such clones in companion animals clinical specimens is worrying both due to therapeutic limitations and public health concerns.

With regards to the body site distribution of isolates carrying different ESBL/pAmpC genes, it is interesting to notice that *bla*_CTX-M-15_ and *bla*_CTX-M-1_ have been found more frequently in samples of the GI tract but also in wound and skin infection isolates, where contamination of the wound due to the licking in dogs is very common. Carriage of ESBL/pAmpC producing *E. coli* of phylogroup B2, D, and F in the GI tract suggests ExPEC intestinal carriage and a potential reservoir of multi-drug resistant ExPEC producing bacteria in animal patients. Previous studies have shown that colonization with *E. coli* of phylogroup B2 in healthy individuals is commonly identified in fecal *E. coli* in healthy humans and animals (Dale and Woodford, 2015; Alonso et al., 2017) and our findings shows that in diseased companion animals, fecal ExPEC isolates can also be ESBL/pAmpC producers. Moreover, we have identified ExPEC ESBL/pAmpC producers amongst clinical isolates from all specimens including UTIs, body fluids/biopsies, skin, and wound infections. This is an important finding for both patient management and infection control in veterinary settings; ExPEC are considered opportunistic colonizing pathogens (Price et al., 2017) and intestinal carriage or skin/wound colonization in immunocompromised patients can lead to infection. Equally important, colonized patients can be a source for hospital dissemination of ExPEC ESBL/pAmpC producers as well as posing a risk for interspecies transmission and a risk for owner and family members. Therefore, infection control measures need to be implemented upon detection of such bacteria to limit their dissemination within veterinary premises, which needs to be coupled by providing owners with information on the significance of isolation of ESBL/pAmpC producers from their pets and provide, for example, hand hygiene advice to reduce transmission risks.

Accurate and rapid laboratory detection of ESBL/AmpC phenotypes is critical for supporting management of patients and infection control in hospitals and clinics. Whilst detection of ESBL/pAmpC *E. coli* colonized patients will be always useful for epidemiology and infection control, distinction between colonization and true infection is critical for patient therapeutic management and reducing unnecessary antibiotic use. In our study, more ESCR isolates were obtained from body sites populated with commensal flora where the role played by the ESCR/ESBL/AmpC producing bacteria in the infection is more difficult to establish as cultures generally result in polymicrobial growth. Conversely, the vast majority of ESCR isolates obtained from normally sterile sites were obtained as pure cultures (77%). Urine samples collected by cystocentesis (where the risk of contamination during collection is the lowest) yielded pure cultures in 91% cases and in most of these (61%) at least one criteria supportive of infection was present. These findings suggest that obtaining an ESCR *E. coli* isolate in pure culture, from a normally sterile site is likely to be clinically significant and warrants antimicrobial treatment. Whenever possible, cytology and Gram-staining can be employed to aid decisions regarding the likely infecting pathogen by revealing the phagocytozed organisms with matching cellular morphology of those obtained from bacterial cultures. Nevertheless, careful assessment of each case is critical for appropriate antimicrobial selection as agents selected to treat the infecting pathogen (other than an ESBL/AmpC producer) may include β-lactam agents and which in turn may select for ESBL/AmpC producers. Repeating bacterial cultures following application of a stringent aseptic technique for specimen collection can also help and should be recommended to distinguish between contamination, transient colonization and true infection.

Our study has shown that a variety of ESBL/pAmpC producing *E. coli* isolates, including ExPEC, can be associated with both colonization and infection in companion animals. However, this work was performed on a bacterial population originating from cases seen in the Small Animal and Equine Referral Hospitals and may not be directly comparable to the general companion animal population. Future prospective studies are necessary to monitor the duration of ESBL/pAmpC producing *E. coli* colonization and identify factors that may trigger the transition from colonization to infection. Better understanding and differentiation of ESBL/pAmpC producing *E. coli* isolates obtained from clinical specimens as true infecting agents or simply transient colonizers will reduce antimicrobial use and therefore preserve the efficacy of currently available antimicrobials and promote antimicrobial stewardship in veterinary practice.

## Data Availability

The raw data supporting the conclusions of this manuscript will be made available by the authors, without undue reservation, to any qualified researcher.

## Author Contributions

AB performed molecular testing, analyzed the data, and wrote the manuscript. AB, GP, and VS reviewed clinical data and performed statistical analysis. AB, AW, CE, FZ, and EM performed phenotypic and molecular characterization of isolates. AB, FZ, EM, and DT analyzed and interpreted the data. DT planned and coordinated the study, analyzed the data, and wrote the manuscript. All authors revised and approved the final version of the manuscript.

## Conflict of Interest Statement

The authors declare that the research was conducted in the absence of any commercial or financial relationships that could be construed as a potential conflict of interest.

## Footnotes

1 https://blast.ncbi.nlm.nih.gov/Blast.cgi
